# Academy of Medicine Notes

**Published:** 1893-04

**Authors:** 


### Academy of Medicine Notes.

Dr. Thomas Lothrop has been unanimously nominated for president, Dr. F. W. Bartlett for trustee, and Dr. E. A. Smith for treasurer, of the Buffalo Academy of Medicine. The election occurs at the annual meeting in June.

The following physicians were elected Fellows of the Academy: Dr. R. R. Ross, Dr. R. Langford Patteson, Dr. C. J. Patterson, Dr. James Stoddart, Dr. Ward B. Whitcomb, (Batavia, N. Y.,) Dr. E. J. Meyer, Dr. L. Denton, and Dr. M. Denton.

The council having wisely decided to select new quarters for the Academy has secured pleasant apartments in the New Market Arcade Building, which will be ready for occupancy May 1st.

The last (March) meeting was one of the best attended meetings of the Academy. We hope to see every chair filled when the Academy is installed in its new home.

A subscription list was started at the meeting held March 21, 1893. Over $200 was subscribed towards furnishing the new rooms. Members who were not present at that meeting may leave their subscriptions with any of the council.

The first amendment to the constitution was adopted at this meeting and reads as follows: Any member of the Academy, in good standing, may be admitted to life membership on payment of $100.

The next stated meeting of the Academy will be held June 27, 1893. The programme will be in charge of the section on Obstetrics and Gynecology. The election of officers and the annual meeting will also be held in June.

At the next meeting of the Section on Surgery of the Buffalo Academy of Medicine to be held April 4, 1893, the following programme will be carried out: Fractures and Competent Surgical Treatment, H. Mynter, M. D.; Legal Aspects and Responsibilities, Mr. W. L. Marcy.

CINCINNATI physicians are agitating the question of consolidation of the various medical societies into an Academy with sections. The Buffalo experiment is proving quite successful and we can recommend the plan to our Cincinnati brethren.

